# Early Vascular Aging in Young and Middle-Aged Ischemic Stroke Patients: The Norwegian Stroke in the Young Study

**DOI:** 10.1371/journal.pone.0112814

**Published:** 2014-11-18

**Authors:** Sahrai Saeed, Ulrike Waje-Andreassen, Annette Fromm, Halvor Øygarden, Marina V. Kokorina, Halvor Naess, Eva Gerdts

**Affiliations:** 1 Department of Heart Disease, Haukeland University Hospital, Bergen, Norway; 2 Department of Clinical Science, University of Bergen, Bergen, Norway; 3 Department of Neurology, Haukeland University Hospital, Bergen, Norway; 4 Department of Clinical Medicine, University of Bergen, Bergen, Norway; Innsbruck Medical University, Austria

## Abstract

**Background:**

Ischemic stroke survivors have high risk of cardiovascular morbidity and mortality even at young age, suggesting that early arterial aging is common among such patients.

**Methods:**

We measured aortic stiffness by carotid-femoral pulse wave velocity (PWV) in 205 patients (69% men) aged 15–60 years with acute ischemic stroke in the prospective Norwegian Stroke in the Young Study. High for age carotid-femoral PWV was identified in the reference normogram.

**Results:**

Patients were on average 49±10 years old, 34% had a history of hypertension and 37% had metabolic syndrome (MetS). In the total study population, higher PWV was associated with history of hypertension (β = 0.18), higher age (β = 0.34), systolic blood pressure (BP) (β = 0.28) and serum creatinine (β = 0.18) and lower high-density lipoprotein (HDL) cholesterol (β = –0.10, all p<0.01) in multivariate linear regression analysis (multiple R^2^ = 0.42, p<0.001). High for age PWV was found in 18% of patients. In univariate analyses, known hypertension was associated with a 6-fold, MetS with a 4-fold and presence of carotid plaque with a 3.7-fold higher risk for high for age PWV (all p<0.01). In multiple logistic regression analysis higher systolic BP (odds ratio [OR] 1.04; 95% confidence interval [CI] 1.02–1.06; p<0.01), history of hypertension (OR 3.59; 95% CI 1.52–8.51; p<0.01), low HDL cholesterol (OR 3.03; 95% CI 1.00–9.09; p = 0.05) and higher serum creatinine (OR 1.04; 95% CI 1.01–1.06; p<0.01) were associated with high for age PWV.

**Conclusions:**

Higher PWV is common in younger and middle-aged ischemic stroke patients and associated with a clustering of classical cardiovascular risk factors.

ClinicalTrials.gov NCT01597453

## Introduction

Risks for recurrent stroke, cognitive problems, cardiovascular morbidity and mortality are high in young and middle-aged patients with ischemic stroke [1], [2]. Current American Heart Association guidelines recommend extensive cardiovascular screening in patients who experience stroke or a transient ischemic attack to prevent recurrent ischemic stroke [3]. In particular identification and management of traditional cardiovascular risk factors, atrial fibrillation and other cardiac sources of systemic cerebral embolism are important in secondary prevention to avoid recurrent ischemic stroke. However, assessment of arterial stiffness is not yet a part of the recommended screening in stroke patients but may contribute to the understanding of the impaired prognosis in patients with a first acute ischemic stroke <50 years old [1], [2].

Carotid-femoral pulse wave velocity (PWV) is a direct measure of central arterial stiffness [4], [5]. Hypertension, atherosclerosis and arterial inflammation all lead to progressive arterial aging which may be diagnosed as increased carotid-femoral PWV for age [6]. Increased arterial stiffness is a well-documented predictor of cardiovascular morbidity and mortality in hypertensive patients as well as in general populations [7], [8]. Arterial stiffness has not been much studied in stroke patients <60 years, but smaller studies in older stroke patients have indicated that increased arterial stiffness is associated with impaired prognosis [9]. The aim of the present study was to assess covariates of increased arterial stiffness in patients ≤60 years with ischemic stroke participating in the Norwegian Stroke in the Young Study (NOR-SYS).

## Methods

### Patient population

The prospective NOR-SYS research program at the Department of Neurology, Haukeland University Hospital, Bergen, Norway includes patients aged 15–60 years, admitted with documented acute ischemic stroke. The rationale, study design, inclusion and exclusion criteria of NOR-SYS have been previously published [10]. Between September 2010 and October 2013, a total of 214 patients were included. Among these, 9 patients were excluded from the present analyses due to presence or history of atrial fibrillation. The subtype of acute ischemic stroke was classified according to the Trial of ORG 10172 in Acute Stroke Treatment (TOAST) criteria into Large Artery Atherosclerosis (LAA), Cardio-Embolic Infarct (CEI), LACunar infarcts (LAC), stroke of Other Determined Etiology (ODE), or stroke of UnDetermined Etiology (UDE) [11].

The study was approved by the Regional Committee for Medical Research Ethics of Western Norway, and conducted in accordance with the Declaration of Helsinki. All patients or their legal representatives signed a written informed consent.

### Cardiovascular risk factors

A standardized questionnaire was used to obtain the patient’s self-reported information about cardiovascular risk factors and medication. Brachial blood pressure (BP) was measured in triplets by the same investigator using a regularly calibrated aneroid sphygmomanometers and appropriate cuff size earlier on the same day of the arterial stiffness assessment [12]. An average of the last two measurements was taken as the brachial BP. Known hypertension was defined as history of hypertension or use of antihypertensive drugs.

Body mass index (BMI) was calculated from body weight in kilograms divided by height in meters squared. Obesity was defined as BMI ≥30 kg/m^2^. Venous blood samples were drawn for analysis of fasting serum lipids and glucose, serum sodium, potassium and creatinine. Hypercholesterolemia was considered present if total serum cholesterol >200 mg/dl, and/or low-density lipoprotein (LDL) cholesterol >116.0 mg/dl. Low high-density lipoprotein (HDL) cholesterol was defined as <40 mg/dl (males) and 50 mg/dl (females) [12].

Metabolic syndrome (MetS) was defined using the modified American Heart Association/National Heart, Lung, and Blood Institute criteria requiring presence of at least 3 of the 5 following criteria: 1) waist circumference ≥102 cm (males) and ≥88 cm (females); 2) triglycerides ≥150 mg/dl; 3) HDL cholesterol <40 mg/dl (males) and 50 mg/dl (females); 4) systolic BP ≥130 mmHg and/or diastolic BP ≥85 mmHg measured at the time of the PWV recording; and 5) fasting blood glucose ≥100 mg/dl [13]. Diabetes mellitus was diagnosed as previously known diabetes, use of anti-diabetic treatment, or fasting blood glucose ≥126 mg/dl [14].

### Pulse wave velocity

**Figure 1:**
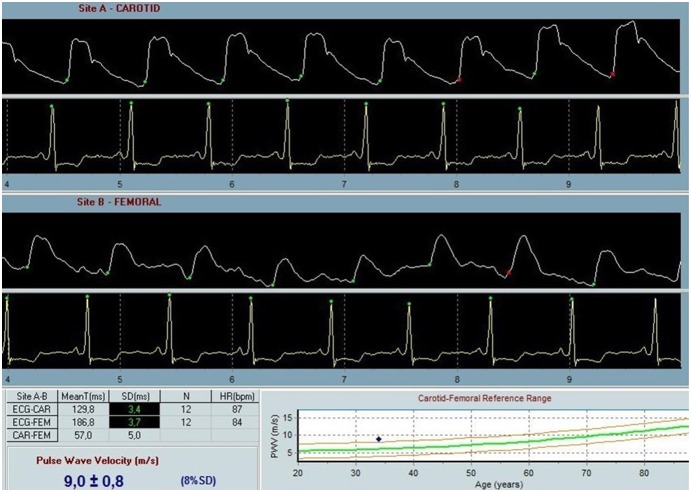
PWV analysis from a study participant with high for age PWV, i.e. PWV above the age-adjusted normative values based on healthy population (16).

Carotid-femoral PWV was measured by the same investigator using a SphygmoCor device (AtCor Medical, Sydney, West Ryde, Australia) under standardized laboratory conditions after the patients had rested for 15 minutes in supine position in a quiet examination room with a stable temperature [15]. All measurements were performed between 8 a.m. and 2 p.m. on average 51 days after the admission for the acute stroke. All patients fasted for at least 2 hours prior to the measurement. During the measurements, speaking or sleeping was not allowed. Pressure pulse waveforms were obtained transcutaneously from the common carotid artery and right femoral artery with simultaneous recording of the electrocardiogram for synchronizing carotid and femoral pulse wave times. Following the inborn quality control indices, optimal curves were obtained from the visual inspection of the waveforms. The foot of the wave was defined at the end of diastole. The transit time was measured between the feet of the 2 waveforms using the intersecting tangent algorithm. The proximal distance between the carotid site and the sternal notch, and the distal distance between the sternal notch and the femoral site were measured precisely. Carotid-femoral PWV was calculated as distance (D) in meters between the 2 recording sites divided by transit time (TT) in seconds (PWV = D/TT) [4]. All PWV values were corrected for mean blood pressure. High for age PWV was taken as PWV higher than age-adjusted normative values based on a large study of 4001 healthy subjects (Figure 1) [16], [17].

### Carotid ultrasound

The carotid intima-media thickness (IMT) was measured by carotid ultrasound as previously published [10]. Carotid plaque was defined as focal IMT >1.5 mm [10], [18].

### Statistics

Statistical analyses were performed using the IBM SPSS statistical program version 21 (IBM, Armonk, New York, USA). Data are presented as mean ± standard deviation for continuous variables and as percentages for categorical variables. The study population was divided into groups with normal and high for age PWV. Comparison of groups was done by Student's t-test and Chi-squared test as appropriate. Comparison between the stroke subtypes was done by analysis of variance (ANOVA) with Scheffe’s post hoc test for PWV and a general linear model with Sidak’s post hoc test for high for age PWV. The variable serum creatinine was skewed and therefore log transformed. Univariate covariates of PWV were identified by Pearson’s correlation coefficient and univariate logistic regression analysis, as appropriate. Independent covariates of PWV were identified in multivariate linear regression analysis and reported as standardized β-coefficients and p-values. Covariates of high for age PWV were identified using uni- and multivariate logistic regression analyses and reported as odds ratio (OR) and 95% confidence intervals (CI). A p value ≤0.05 was considered statistically significant in both uni- and multivariate analyses.

## Results

### Total study population

The total population was on average 49±10 years old and included 69% men, 37% with MetS, 34% with history of hypertension, 13% with diabetes mellitus and 11% with carotid plaques (Table 1). Common cardiovascular risk factors like hypertension, diabetes mellitus, obesity, smoking and hypercholesterolemia clustered in the study population, and 58% of patients had at least 2 of these risk factors. PWV was numerically higher in patients with LAA, LAC and UDE stroke subtypes, but differed significantly only between UDE and CEI stroke subtypes (p<0.01) (Table 2). Among the subpopulation of 134 (65%) patients who underwent transesophageal echocardiography, 22% had a patent foramen ovale (PFO). Among patients with PFO, 71% were classified as CEI, 2% LAA, 5% LAC and 22% as UDE stroke subtypes.

**Table 1:** Baseline characteristics of the study population.

	Total population	Normal for age PWV	High for age PWV
Number of patients, n	205	167	38
Age, y	49±10	48±10	52±7**
Male sex, %	69	66	82*
Metabolic syndrome, %	37	31	64**
Obesity, %	24	20	42**
Smoking, %	46	46	47
Diabetes mellitus, %	13	10	29**
Antidiabetic treatment, %	7	4	16*
Known hypertension, %	34	26	68**
Antihypertensive treatment, %	22	19	39**
Systolic BP, mmHg	133±19	129±17	148±21**
Diastolic BP, mmHg	81±11	79±10	88±14**
Mean BP, mmHg	99±13	96±12	108±15**
Pulse pressure, mmHg	52±13	50±12	60±13**
PWV, m/sec	8.0±1.9	7.3±1.2	11.1±1.6**
Carotid plaque, %	11	8	24**
Creatinine, mg/dl	0.85±0.39	0.79±0.15	1.07±0.85
Total cholesterol, mg/dl	213±46	212±42	224±54
HDL cholesterol, mg/dl	54±19	54±19	46±12**
LDL cholesterol, mg/dl	143±42	139±39	151±46
Triglycerides, mg/dl	142±89	133±80	204±115**
Fasting blood glucose, mg/dl	103±25	100±18	118±40*

Data are mean±SD or percentage.

*p<0.05.

**p<0.01.

**Table 2:** PWV and prevalence of high for age PWV in stroke subtypes according to the TOAST classification.

	LAA	CEI	LAC	ODE	UDE	p value
Number of patients, n	12	50	42	23	78	
PWV, m/s	8.8±1.7	7.2±1.7	8.3±1.8	7.7±1.4	8.4±2.1*	<0.01
High for age PWV, %	25	12	21	17	20	ns

*p = 0.01 vs CEI. LAA: large-artery atherosclerosis; CEI: cardio-embolic infarct; LAC: lacunar infarct; ODE: stroke of other determined etiology; UDE: stroke of undetermined etiology.

In the total study population, presence of carotid plaque was associated with higher PWV (β = 0.25, p<0.001). Univariate covariates of PWV in the total study population are presented in Table 3. In the overall study population, history of hypertension (β = 0.18), higher age (β = 0.34), systolic BP (β = 0.28) and serum creatinine (β = 0.18), and lower HDL cholesterol (β = –0.10, all p<0.05) were identified as independent covariates of higher PWV in multivariate analysis (Table 4). In a secondary model, replacing serum HDL cholesterol and history of hypertension with presence of MetS, MetS was associated with higher PWV independent of age, systolic BP and presence of carotid plaque (Table 4). No association of the time interval between the acute stroke and the PWV recording, or between new-onset or intensified antihypertensive drug treatment during hospitalization for the acute stroke and PWV was found.

**Table 3:** Covariates of PWV in univariate analyses in the total study population.

Variables	Pearson's correlation coefficient	p value
Age, y	0.47	<0.001
Systolic BP, mmHg	0.46	<0.001
Diastolic BP, mmHg	0.35	<0.001
Pulse pressure, mmHg	0.39	<0.001
Mean BP, mmHg	0.44	<0.001
Weight, kg	0.32	<0.001
Body mass index, kg/m^2^	0.28	<0.001
Waist circumference, cm	0.42	<0.001
Total cholesterol, mg/dl	0.14	<0.05
LDL cholesterol, mg/dl	0.19	<0.01
HDL cholesterol, mg/dl	–0.25	<0.001
Triglycerides, mg/dl	0.29	<0.001
Serum creatinine, mg/dl	0.28	<0.001
Fasting blood glucose, mg/dl	0.29	<0.001
HbA1c, %	0.37	<0.001

**Table 4:** Independent covariates of higher PWV in multivariate linear regression analyses.

	Model 1*		Model 2**	
Variables	Beta	p value	Beta	p value
Age, y	0.34	<0.001	0.38	<0.001
Systolic BP, mmHg	0.28	<0.001	0.32	<0.001
Known hypertension	0.18	<0.01	n.a.	n.a.
Carotid plaque	0.05	0.39	0.09	0.10
Metabolic syndrome	n.a.	n.a.	0.15	<0.05
Serum creatinine, mg/dl	0.18	<0.01	0.12	<0.05
HDL cholesterol, mg/dl	–0.10	0.05	n.a.	n.a.

n.a. = Not applicable. *multiple R^2^ 0.42, p<0.01; **multiple R^2^ 0.40, p<0.01*.

### High for age PWV

High for age PWV was found in 18% of patients, and among these 77% had a PWV >10 m/sec, the current guideline recommended cut-off value for high PWV [12]. Compared to patients with normal for age PWV, patients with high for age PWV were older, dominated by males, had higher BP, serum creatinine, fasting blood glucose and serum triglycerides and lower plasma HDL cholesterol, as well as had more often known hypertension, diabetes mellitus, obesity, MetS and carotid plaques (all p<0.05) (Table 1). Clustering of cardiovascular risk factors like hypertension, diabetes mellitus, obesity, smoking and hypercholesterolemia was particularly high in this group: 87% of patients with high for age PWV had at least 2 and 51% had at least 3 of these risk factors. The prevalence of high for age PWV did not differ between different TOAST subtypes of stroke (Table 2). In univariate logistic regression analyses, known hypertension was associated with a 6-fold (OR: 6.25 [95% CI 2.90–13.04], p<0.001), MetS with a 4-fold (OR: 4.10 [95% CI 1.87–8.99], p<0.001) and presence of carotid plaque with a 3.7-fold (OR: 3.70 [95% CI 1.45–9.45], p<0.01) higher risk for high for age PWV. In multivariate analyses adjusting for systolic BP and serum creatinine, having history of hypertension or MetS both were independently associated with presence of high for age PWV in different models (Table 5).

**Table 5:** Independent covariates of high for age PWV in multiple logistic regression analyses.

	Model 1		Model 2	
Variables	OR (95% CI)	p value	OR (95% CI)	p value
Systolic BP, mmHg	1.04 (1.02–1.06)	<0.01	1.05 (1.02–1.07)	<0.01
Known hypertension	3.59 (1.52–8.51)	<0.01	n.a.	n.a.
Carotid plaque	1.38 (0.45–4.27)	0.57	3.47 (1.05–11.44)	<0.05
Metabolic syndrome	n.a.	n.a.	2.59 (1.06–6.36)	<0.05
Low HDL-cholesterol	3.03 (1.00–9.09)	0.05	n.a.	n.a.
Serum creatinine, mg/dl	1.04 (1.01–1.06)	<0.01	1.05 (1.02–1.08)	<0.01

n.a. = Not applicable.

## Discussion

Few studies have so far reported on arterial stiffness in ischemic stroke patients [9], [19], [20]. To our knowledge, the present study is the first to report on arterial stiffness and the association with clustering of cardiovascular risk factors among young and middle-aged ischemic stroke patients. In particular, high for age PWV was found in 18% of patients and associated with higher systolic BP, unfavorable serum lipid profile, history of hypertension and presence of MetS and carotid plaques, reflecting atherosclerosis detected by ultrasound.

The prevalence of high for age PWV in our population was comparable to that reported from the general Danish population in subjects, and therefore lower than expected [8]. Of note, 23% of patients with high for age PWV had a PWV <10 m/s, the guideline-suggested threshold value for diagnosing target organ damage.

The overall prevalence of hypertension in our population was high, and hypertension was a main covariate of higher PWV in young and middle-aged ischemic stroke patients in the present study. Hypertension is a well-known risk factor for stroke and increased arterial stiffness [7], [21]. From the large Campania Salute Network including more than 6000 hypertensive patients, higher arterial stiffness was associated with higher prevalence of carotid atherosclerotic plaques identified by ultrasound [22]. The present study extends this association to young and middle-aged stroke patients, by confirming the independent association of high for age PWV and presence of pre-cerebral atherosclerosis.

Another striking finding in the present study was the clustering of metabolic risk factors among young and middle-aged patients with ischemic stroke, and in particular those with high for age PWV. More than 50% of patients in this subgroup had at least 3 modifiable cardiovascular risk factors. This finding points to the vast potential for both primary and secondary prevention to avoid ischemic stroke as well as recurrent stroke in young and middle-aged patients. Of note, only 58% of patients with high for age PWV and known hypertension in the present study population received antihypertensive treatment at the time of the ischemic stroke.

Presence of MetS was associated with increased arterial stiffness independent of systolic BP and history of hypertension in the present study, adding to findings previously reported in older ischemic stroke patients [19]. The amplification of age-associated increase in arterial stiffness by MetS has been reported in a general population using carotid ultrasound imaging to derive arterial compliance [23]. Furthermore, an association between MetS and subclinical atherosclerosis was previously reported in the Cardiovascular Risk in Young Finns Study including 2163 Finnish adults below 40 years of age [24]. In their study, presence of MetS was also associated with increased carotid artery intima-media thickness and reduced carotid arterial compliance assessed with ultrasound [24].

Low HDL cholesterol was associated with a 3-fold increased presence of high for age PWV independent of hypertension and systolic BP level in the present study. In several population-based studies, lower HDL cholesterol has been associated with arterial stiffness [25], [26].

Increased carotid-femoral PWV has also been documented as an independent predictor of mortality among diabetic patients [27]. The overall prevalence of diabetes in NOR-SYS was 13%, but twice as common in the high for age PWV group. The shared effect of endothelial dysfunction, chronic low-grade inflammation, glycation product formation and changes in structure of elastin and collagen fibers in the arterial wall are all pathophysiological mechanisms suggested to contribute to the increased arterial stiffness in diabetes [28]. Of note, among patients with high for age PWV in the present study population, only half of the diabetic patients received glucose lowering agents at the time of the ischemic stroke, pointing to the consequences of poor primary cardiovascular prevention.

In conclusion, high for age PWV was found in 18% in this prospective study of young and middle-aged ischemic stroke patients, and particularly associated with the presence of hypertension, MetS and carotid plaques. The clustering of modifiable cardiovascular risk factors in young and middle-aged stroke patients underline the vast potential for improving prognosis in these patients by implementation of effective secondary prophylaxis. Whether patients with high for age PWV are at particular risk for future cardiovascular events should be investigated in a larger study.
